# Examining the Role and Strategies of Advocacy Coalitions in California’s Statewide Sugar-Sweetened Beverage Tax Debate (2001-2018)

**DOI:** 10.1177/08901171231201007

**Published:** 2023-09-20

**Authors:** Kesia K. Garibay, Nancy J. Burke, A. Susana Ramírez, Denise D. Payán

**Affiliations:** 1 Department of Public Health, School of Social Sciences, Humanities and Arts, 33244 University of California Merced, Merced, CA, USA; 2 Department of Health, Society, and Behavior, Program in Public Health, 8788 University of California Irvine, Irvine, CA, USA

**Keywords:** advocacy coalitions, public policy, sugar-sweetened beverage, tax, California

## Abstract

**Purpose:**

California’s failed attempts to enact a statewide sugary beverage tax presents an opportunity to advance understanding of advocacy coalition behavior. We investigate the participation of advocacy coalitions in California’s statewide sugar-sweetened beverage (SSB) tax policy debate.

**Design:**

Document analysis of legislative bills and newspaper articles collected in 2019.

**Setting:**

California.

**Method:**

A total of 11 SSB tax-related bills were introduced in California’s legislature between 2001-2018 according to the state’s legislative website. Data sources include legislative bill documents (n = 94) and newspaper articles (n = 138). Guided by the Advocacy Coalition Framework (ACF), we identify advocacy coalitions involved in California’s SSB tax debate and explore strategies and arguments used to advance each coalitions’ position.

**Results:**

Two coalitions (public health, food/beverage industry) were involved in California’s statewide SSB tax policy debate. The public health coalition had higher member participation and referred to scientific research evidence while the industry coalition used preemption and financial resources as primary advocacy strategies. The public health coalition frequently presented messaging on the health consequences and financial benefits of SSB taxes. The industry coalition responded by focusing on the potential negative economic impact of a tax.

**Conclusion:**

Multiple attempts to enact a statewide SSB tax in California have failed. Our findings add insight into the challenges of enacting an SSB tax considering industry interference. Results can inform future efforts to pass evidence-based nutrition policies.

## Introduction

Sugar-sweetened beverage (SSB) consumption is a major contributor to the obesity epidemic^1^ and diet-related health disparities, including type 2 diabetes, cardiovascular disease, and dental caries.^2-5^ In 2015, 63% of U.S. adults consumed at least one SSB per day; Blacks, Latinos, low socioeconomic households, and people with less than a high school education consumed SSBs most frequently.^6^ Taxing SSBs is an evidence-based policy approach to reduce consumption,^7,8^ lower healthcare costs, and generate significant health gains.^9^

Decades of evidence from tobacco and alcohol tax research demonstrate that excise taxes levied on a per-unit basis are more effective than taxes applied to a broader range of goods and services with a greater impact on relative prices.^10^ Growing evidence of the efficacy of SSB taxes at the municipal level in the U.S. and at the country level globally^11-13^ has motivated several states to consider SSB excise taxes.^14^ Berkeley, California enacted the country’s first SSB excise tax in 2014; the tax has been credited with a decline in SSB sales and increased sales of water/non-taxed drinks.^12^ The tax narrowed disparities: SSB consumption decreased by 21% in low-income neighborhoods (2014-2015) compared to a 4% increase in comparison cities.^7^ Similar effects have been documented elsewhere in the U.S.^15^ and other countries (e.g., Mexico,^11^ South Africa,^13^ Finland, France, Belgium, and Portugal^16^). Despite evidence of the policy's effectiveness, to date, only local U.S. jurisdictions have adopted SSB taxes.

SSB taxes are heavily opposed by the beverage industry, which pours significant resources into anti-tax lobbying.^17^ Public health advocates thus face strong opposition to the adoption of SSB taxes. Nonetheless, the strategies they have used to influence local public policy debates can be instructive. For example, in Berkeley, the pro-SSB tax campaign successfully leveraged social media to frame the debate using public health terms, despite the American Beverage Association spending millions of dollars to oppose the tax.^18^ How these strategies were enacted, and by whom, can be examined using the Advocacy Coalition Framework (ACF). Advocacy coalitions are defined as groups of actors with shared beliefs who: engage in political strategies and debates, impact policy by engaging legislators, and leverage resources to advance their position. Studies on state menu labeling^19^ and tobacco^20^ policy debates have identified two main coalitions: public health and industry, who may be similar to the types of coalitions involved in SSB tax debates. While a pro-SSB tax public health coalition may consist of policymakers, public officials, public health advocates, and researchers,^17^ an industry coalition may include industry actors and lobbyists.^21^ Despite the potential to inform future public debates, limited research has examined the role of advocacy coalitions and their strategies in SSB tax policymaking debates, including the types of arguments leveraged in support or opposition to a specific policy position.

## Purpose

This article examines the role and involvement of advocacy coalitions in California’s SSB tax policy debate. The study’s objectives were to: (1) identify advocacy coalition groups, leaders, and participants; (2) examine their strategies; and (3) explore policy arguments to support or oppose statewide SSB tax legislation in California.

## Methods

**Figure 1.**
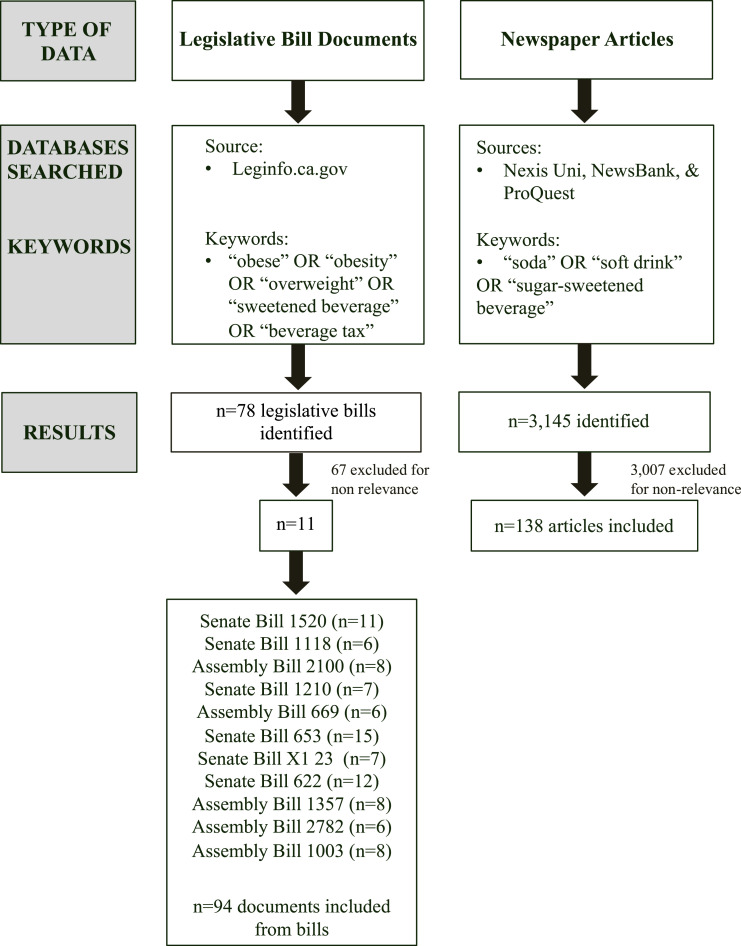
Search process for sugar-sweetened beverage tax legislative material and related newspaper articles in California (1999-2018).

This qualitative study used document analysis^22^ to investigate the types of advocacy coalitions involved in California’s statewide SSB tax debate and their related activities. Document analysis was selected as the analytical approach since it is useful for understanding policy content and processes over time.^23,24^ Data sources include California legislative bill documents and newspaper articles (Figure 1).

The Advocacy Coalition Framework (ACF) guided data collection and analysis of written materials. The ACF posits that advocacy coalitions are bound by a belief system comprising common perceptions and value priorities where individuals and organizations with similar policy belief systems engage in coordinated activities to promote a position. The ACF distinguishes between three sets of beliefs: (1) deep core beliefs that drive a coalition's position; (2) policy core beliefs, focused on strategies and policy positions to advance deep core beliefs; and (3) secondary beliefs focusing on the administration and implementation of the policy.^25,26^ In addition to investigating whether expressed coalition arguments reflected these types of beliefs, we consider long-term coalition opportunity structures, coalition membership, policy advocacy strategies, and short-term constraints and resources of subsystem actors.

### Data Collection: Legislative Bill Documents

In 2019, we systematically searched for SSB tax bills introduced in California’s legislature between 1999-2018 using the state’s publicly available legislative website using “sweetened beverage” and “beverage tax” as key words. This study was part of a parent study to develop a comprehensive state obesity policy database; as such, the initial search terms also included “obese”, “obesity”, and “overweight”.

After excluding bills due to non-relevance (e.g., SSB warning labels, alcoholic beverage tax), 11 bills were identified, which were associated with n = 94 legislative documents. Legislative documents were legislative bills with background information including a bill’s history, status, text, votes, analysis, and amendments. To ensure the search was comprehensive, in 2023, we conducted additional searches for bill documents published between 1999-2018 using the following terms: soda, soft drink, sugar-sweetened beverage, sugary drink tax, sugary beverage tax, and sugar sweetened beverage tax. However, we did not identify any new SSB tax bills.

### Data Collection: News Coverage

We also collected newspaper articles reporting on the state’s SSB tax policy proposals and debates in 2019. Articles were identified using the online archives Nexis Uni, NewsBank, and ProQuest with key terms “soda” OR “soft drink” OR “sugar-sweetened beverage” in the title or text. Inclusion criteria were articles published: in California, in English, and between 1999-2018. We found 3145 articles, excluding 3007 which were duplicates or covered only local SSB taxes in California, non-tax policies (i.e., warning labels), or the health effects of diet soda. The final sample included n = 138 newspaper articles.

### Data Analysis

Legislative documents and newspaper articles were uploaded into the qualitative data analysis software Atlas.ti for coding and analysis. For each bill, we identified the bill type, author(s), key policy details, advocacy strategies used, type of research evidence used (i.e., type 1 includes epidemiological data to describe the magnitude and severity of a public health problem, type 2 on the relative effectiveness of specific interventions and type 3 focuses on the context, design and implementation of an intervention), policy precedence (i.e., reference to other similar taxes), and supporting or opposing arguments. The study’s qualitative codebook included themes identified a priori based on the research questions, ACF constructs,^25^ policy arguments,^27-30^ and prior research.^31^ Of note, advocacy coalition arguments are a type of strategy (i.e., communication) that coalitions can use to advance their policy position therefore the arguments codes were considered a sub-theme.

We pilot tested the codebook on each type of legislative document and three newspaper articles. We made changes to the codebook based on these results and used the final version to code all content.^32^ No new related themes emerged during the full coding process. Documents on the bill history, status, and any votes were used to develop a timeline of legislative events. We also conducted a summary analysis of legislative content and used Microsoft Excel to calculate descriptive statistics. The most frequently mentioned advocacy coalition strategies and arguments are included in the results; each was mentioned at least three different times and were often present in multiple types of documents (i.e., legislative material and newspaper articles).

## Results

### Summary of SSB Tax Bills (2001-2018)

**Figure 2.**
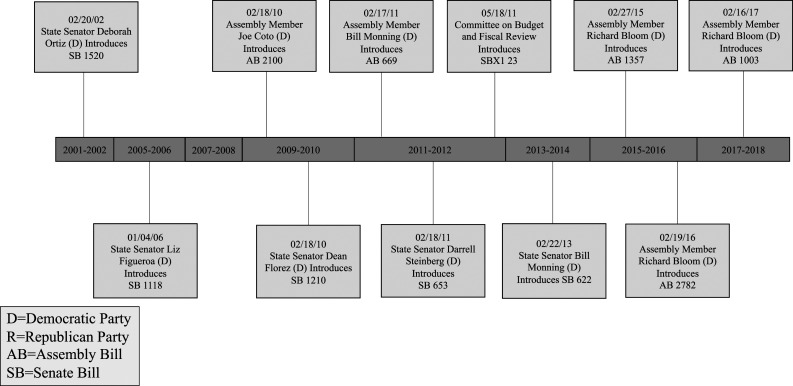
Timeline of sugar-sweetened beverage tax bills introduced in California between 2001 and 2018.

The California state legislature introduced a total of 11 SSB tax bills between the 1999-2000 and 2017-2018 legislative sessions (see Figure 2), six were Senate Bills (SB) and five were Assembly Bills (AB). Between 2011-2012, the highest number of bills were introduced (three bills)— signaling higher interest and policy activity. Eight failed to leave their committee; and, ultimately, all 11 bills failed.

While the specific components of the bills differed, all bills proposed a tax on beverages with added sugar and mentioned a specific tax percentage or rate. In 2001, SB 1520 was the first introduced, proposing a surtax upon every distributor, manufacturer, or wholesale dealer at a rate of $2 per gallon of soft drink/simple syrup and $0.21 per gallon of bottled soft drinks or those produced from powder. Among the remaining bills: two included a tax of $0.01 per teaspoon of added caloric sweetener (AB 2100, SB 1210), four a rate of $0.01 per fluid ounce (AB 669, SB 653, SBX1 23, SB 622), three a rate of $0.02 per fluid ounce (AB 1357, AB 2782, AB 1003), and one a 2% per carbonated beverage rate (SB 1118).

In nine of the bills, the State Board of Equalization was set to administer the tax; however, the mechanisms for enforcement were absent. All of the bills aimed to use the proposed tax revenue to support California’s schoolchildren. Specifically, two of the bills allowed for school districts to tap into the SSB tax revenue in certain instances. Eight of the bills aimed to use the funds to address childhood obesity in school or early childcare settings; of these, six bills designated the tax to fund childhood obesity prevention programs. Three included a secondary policy to establish or modify an existing advisory group (e.g., commission, committee, council, task force) to address obesity.

### Summary of Media Attention

**Figure 3.**
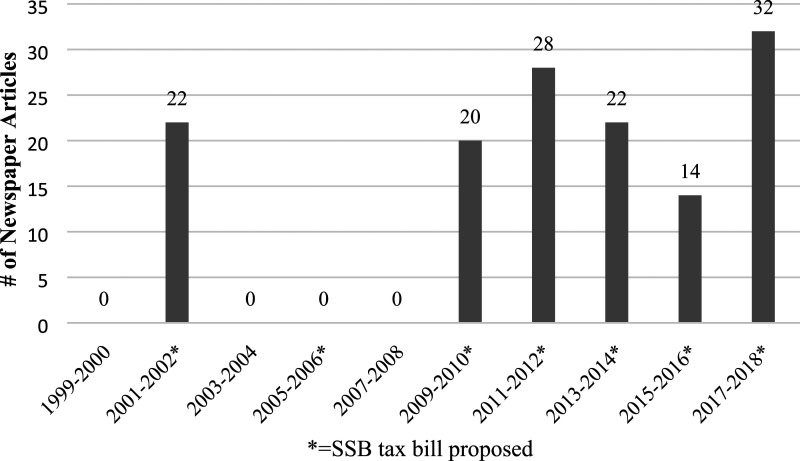
Newspaper articles featuring California’s proposed legislative bills to tax sugar-sweetened beverages, 1999-2018 (n = 138).

The SSB excise tax bills garnered some media attention. From 2001-2018, an average of 14 newspaper articles per 2-year legislative session were published on California’s SSB tax debate (Figure 3). In 2002, when SB 1520 was first introduced, 22 newspaper articles were published within the leglislative cycle (2001-2002). The highest level of media activity occurred in 2017-2018 when a preemption bill (AB 1838) was enacted (32 articles), preemption occurs when a higher level of government limits the authority of lower levels to enact laws.^33^ The second highest level of media activity occurred in 2011-2012 when three SSB tax bills were proposed, and 28 articles were published.

### Characterizing the Coalitions: Public Health and Industry Coalitions’ Leaders and Members

Grounded in the Advocacy Coalition Framework, our analytical approach to identify core organizational leaders and members in terms of their ongoing participation over time in the policy debate provides support that there were two major competing coalitions involved in the debate over SSB taxes in California: a public health coalition, comprised of public health advocacy and healthcare professional organizations, and an industry coalition, comprised of food and beverage trade and lobbying organizations. A single Assemblymember, Richard Bloom introduced three of the bills; Assemblymember Bill Monning introduced two of the bills, the remaining six bills were introduced by six different state legislators, all Democrats, representing regions around the state.

We identified a total of 22 core organizational leaders: 12 for the public health coalition and ten for the industry coalition. Table 1 provides a list of core organizations. We defined core organizational leaders as organizations who: (1) sponsored/cosponsored at least two SSB tax bills, or (2) visibly supported/opposed an SSB tax bill for at least 5 of 11 bills identified.

**Table 1.** Level of Involvement, Criteria Description, and Organizations Listed for Core Organizational Leaders Involved in California’s SSB tax Policy Debate Between 2001-2018.

Level of involvement	Criteria Description	Organization
Sponsored or cosponsored at least 2 of the eleven bills	Sponsored 2 bills and cosponsored 1 bill	• Public health advocates (formerly the California center for public health advocacy)
	Cosponsored 3 bills	• American heart association
		• Latino coalition for a healthy California
		• California dental association
	Cosponsored 2 bills	• American diabetes association
		• California black health network
		• Public health institute
		• Roots of change
Visibly supported an SSB statewide tax in legislative documents for at least 5 of the eleven bills identified	Supported 6 or more bills	• American federation of state, county, municipal employees, state council
		• California primary care association
	Supported 5 bills	• California food policy advocates
		• Center for science in the public interest
Visibly opposed an SSB statewide tax in legislative documents for at least 5 of the eleven bills identified	Opposed 9 or more bills	• California grocers association
		• California retailers association
		• California restaurant association
	Opposed 8 bills	• California chamber of commerce
		• California manufacturers and technology association
	Opposed 6 bills	• California automatic vendors council
		• California Nevada soft drink association
		• California taxpayers association
	Opposed 5 bills	• Grocery manufacturers association
		• Howard jarvis taxpayers association

**Figure 4.**
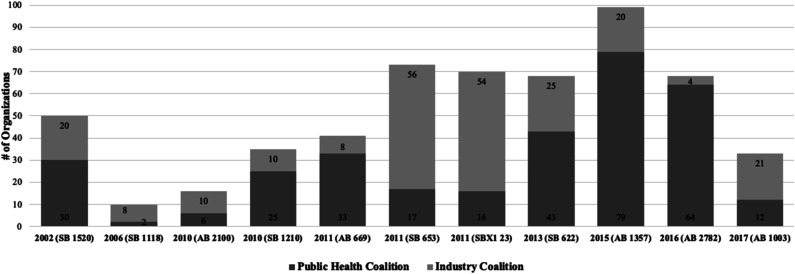
Number of organizations in each advocacy coalition by SSB tax bill and year in California (2002-2017).

In terms of coalition membership, Figure 4 shows the number of organizations that supported or opposed an SSB tax bill. Coalition membership, was defined as organizations sponsoring or opposing a bill and named in legislative documents. On average, 33 public health organizations supported a SSB tax bill compared to 24 industry organizations who opposed a bill per legislative cycle. The public health coalition began with more organizations (30) who publicly supported SB 1520 in 2002 compared to the 20 industry organizations who opposed a bill. Between 2002-2015, the public health coalition more than doubled in size while the industry coalition remained consistent, with some fluctuations.

### Public Health Coalition’s Advocacy Strategies: Use of Research Evidence and Policy Arguments

Public health coalition leaders primarily used communication strategies to advance their policy position. They frequently mentioned and cited research evidence to argue in favor of a statewide SSB tax, e.g., mentioning epidemiological data on the severity of the obesity epidemic to justify a tax. An often-mentioned study found that, although the percentage of children (<12 years) who drink at least one SSB per day declined between 2005-2012, consumption increased among adolescents.^34^ Adults who drank a daily SSB were also said to be 27% more likely to be overweight compared to their counterparts.^35^ Public health advocates frequently mentioned the health consequences of excess SSB consumption (i.e., obesity, diabetes, dental diseases) while arguing in favor of an SSB tax. In 2018, advocates said:

There is clear and compelling body of evidence [that] now shows a strong relationship between consumption of sugary drinks and type 2 diabetes, obesity, and heart, liver, and dental diseases. In fact, every year, 40,000 deaths in the U.S. are attributed to heart problems caused specifically by consuming too many sugary beverages.^36^

Related to other types of research evidence, public health advocates also referenced local SSB taxes and the effectiveness of Berkeley’s policy.^12^ They frequently mentioned state tobacco taxes to exemplify how an SSB tax might decrease the purchase of a targeted product and provide revenue for prevention programs. A 2015 article quoted public health coalition leader, then State Senator Bill Monning (D), on the potential health benefits of a tax: “We see a lot of the data that shows the adverse consequences of sugar-sweetened beverages, but what's notable is [the study] goes straight to the correlation between a sugar-sweetened beverage tax, and consumption and better health outcomes.”^37^ Newspaper articles and legislative documents described how the SSB tax could provide local revenue for obesity and diabetes prevention and reduction programs. The tax was also said to potentially reduce the amount of healthcare spending on obesity-related expenses. In a 2011 *Times-Herald* article, Assemblymember Bill Monning (D) stated,

We cannot afford to sit back while the childhood obesity crisis overwhelms our healthcare system and shortens our children's lives. The public is already paying for costs associated with unhealthy lifestyles and a major contributor to this is the increased consumption of sweetened beverages. A tax on sugary drinks will help to address this growing problem and can be a valuable tool in a broader public health campaign.^38^

Public health advocates also expressed concern about the disproportionate impact of SSB consumption on marginalized groups, including low-income, African American, and Latino populations, who were more likely to reside in unhealthy environments. A 2009 article quoted San Joaquin County Public Health Services Director Bill Mitchell,

County adolescents may drink more soda than their peers in other counties for social reasons. Education and poverty play a part. You have children living in areas where they don’t have access to health-food stores and may not have access to information to teach them healthier eating habits.^39^

### Industry Coalition Advocacy Strategies: Preemption, Financial Resources, and Policy Arguments

In contrast to the public health coalition, the industry coalition used a combination of policy strategy and anti-tax arguments. The core policy strategy used by the industry coalition in newspaper articles was the *preemption* law, AB 1838, to oppose and prevent the enactment of any new SSB taxes. In 2018, AB 1838 was enacted imposing a 12-year ban on local soda taxes and removed a statewide ballot measure. The ballot measure, an effort led by the Service Employee International Union (SEIU) and American Beverage Association, would have required cities and counties to obtain a supermajority (two-thirds)^40^ approval from voters to raise new taxes, making it difficult to enact new taxes and raise revenue. The preemption law prohibited the imposition, increase, levy, and collection, or enforcement by a local agency of any tax, fee, or other assessment on groceries until January 1^st^, 2031, allowing for the continuance of local taxes or fees enacted before 2018 (e.g., Berkeley’s SSB tax). The law defined groceries as any raw or processed food or beverage including its packaging or container, or any ingredient thereof, intended for human consumption.^41^

A quid pro quo arrangement for AB 1838's passage may have occurred since beverage industry representatives offered to withdraw a statewide ballot initiative in exchange for lawmaker support.^40,42^ A *Sacramento Bee* article mentioned a dinner meeting between California Governor Jerry Brown (D) and beverage industry representatives:

Both the governor and the American Beverage Association said the dinner had nothing to do with the proposed soda tax ban, though they declined to answer questions about what they discussed. But public health advocates, who have successfully pushed for taxes on sugary drinks in California cities, decried the meeting as evidence of soda companies' undue influence at the Capitol and the proposed ban as a "sweetheart deal" to protect their profits.^43^

However, no specific records of the conversation exist, and both the governor and beverage industry representatives said the dinner had nothing to do with the proposed soda tax ban.^43^

In addition to preemption, the industry coalition leveraged financial resources. A 2018 article mentioned the impact of financial resources on AB 1838 votes: “[State legislators] reluctantly voted to impose the moratorium because the ballot measure, for which signatures gathered by a political campaign financed by more than $7 million from the beverage industry.”^44^ A report also mentioned the soft drink industry spent $11.8 million statewide on political campaigns and lobbying efforts in 2017-2018.^45^

SSB tax opponents frequently argued that individual choice/responsibility and lack of physical activity were drivers of obesity, with parents named as ideal authority figures responsible for children’s SSB access. In 2011, Assemblymember Diane Harkey (R) said, "I think parents need to step up and they're just not. We need to get parents back involved rather than trying to limit consumption through a tax.”^46^

Opponents often said an SSB tax unfairly targeted soft drinks when multiple factors contributed to obesity and chronic illness (e.g., sedentary behavior). In the AB 1357 bill analysis, Californians for Food and Beverage Choice stated,

Obesity and diabetes are complex health issues that have myriad contributing factors including genetics, physical activity, and calorie intake from all sources – not just beverages. As a result, it is unfair and inaccurate to portray SSBs as the main culprit.

Opponents argued a tax would be an economic burden for consumers and distributors, resulting in increased prices. SSB distributors were said to operate at small profit margins; therefore, a tax could harm profits and operations, potentially leading to job losses and business closures for small retailers and new businesses.

Similar to the public health coalition, the industry coalition mentioned an impact on marginalized communities. However, instead of the effect of SSB consumption, they focused on the tax policy instrument and claimed taxation could penalize disadvantaged, low-income communities. Distributors were expected to pass the tax to consumers leading to increased household food budgets. In 2018, an American Beverage Association spokesperson said, “Our aim is to help working families by preventing unfair increases to their grocery bills.”^47^

## Discussion

California’s statewide SSB tax policy debate and experience between 2001-2018 reveals numerous lessons that can inform future SSB tax campaigns. As prior debates about state tobacco taxes, nutrition policy, and SSB taxes passed elsewhere,^48^ we found a public health coalition and industry coalition were involved in California’s SSB tax policy debate. Comparable coalition leaders were involved in California’s menu labeling debate,^31^ namely, the American Heart Association (AHA), and California Center for Public Health Advocacy for the public health coalition and the California Restaurant Association (CRA) for the industry coalition, thus signaling the active and important role of state-level organizations in nutrition legislation advocacy.

The ACF posits that coalitions use strategies and resources to influence the policymaking process. In California, the public health coalition primarily used communication strategies to convey scientific information to advance an SSB tax. This finding differs from California’s menu labeling debate, where research evidence focused on describing the scope of the public health problem of obesity rather than the effectiveness of the policy instrument.^19^ Our study reveals that the existence of policy effectiveness research from local SSB taxes was insufficient as a basis to enact a statewide tax—an important lesson for SSB tax advocates. Instead, other types of advocacy strategies may need to be leveraged in addition to research evidence to increase the likelihood of enacting a statewide SSB tax in the U.S. Examples of other advocacy strategies include early stakeholder engagement (including retailers), public opinion polls, or coupling the tax revenue to support another underfunded area on the policy agenda (e.g., prekindergarten)^49^ to expand support. Of note, it is possible that the effectiveness of evidence-based arguments to support a new tax may differ compared to other policy instruments that are perceived to be less authoritative or onerous on economic development (e.g., nutrition warning labels, media campaigns).

Alternatively, the industry coalition, led by the beverage industry, primarily used preemption and financial resources, in addition to messages about higher grocery costs. While California’s preemption law (AB 1838) was enacted in 2018 and mentioned in media articles, this law was not identified in the legislative search or included in the legislative document analysis because it focused on grocery taxes on carbonated and noncarbonated nonalcoholic beverages. In recent years, the food and beverage industry has increasingly used preemption to oppose SSB taxes and curtail local authority, preventing policies to improve health.^50^ In California, the beverage industry successfully advocated for a 2018 preemption law with a moratorium that prohibited cities from enacting new SSB local taxes through 2031. Since 2017, Arizona, Michigan, and Washington passed laws preempting local SSB tax policies; California and Washington were the only two that grandfathered existing taxes.^51^ In terms of strategy, the beverage industry has pursued state preemption using front groups and trade associations, lobbying key policymakers, inserting preemptive language into other legislation, and issuing legal threats and challenges.^52^

California’s preemption example and framing by the industry coalition can provide valuable insight for advocates strategizing to pursue evidence-based nutrition policies. Public health advocates state that beverage companies have obscured SSB tax policies by framing them as “grocery” taxes,^53^ which may be viewed as a scare tactic to elicit concern from low-income and communities of color.^54^ Industry coalition members said distributors would pass the tax on consumers in the form of higher taxes on all groceries—similar to arguments present in Berkeley’s local SSB tax debate (i.e., tax will raise grocery bills because store owners would increase the cost of other food products to offset the cost of soda). However, there was no evidence of higher grocery bills due to Berkeley’s SSB tax.^12^

The ACF mentions coalitions’ ability to translate beliefs into policy determines their success.^55^ Fundamental beliefs that drove the public health coalition’s activities include highlighting the link between chronic disease and SSB consumption, which was a priority in national health policy documents and media of 13 countries who implemented a SSB tax.^48^ Additionally, in India, Ireland, South Africa, Thailand and the United Kingdom, tooth decay was mentioned as a health problem associated with SSB consumption in the media.^48^ This message was not commonly included in the media in California’s SSB tax debate. Pro-tax messaging for local SSB taxes has been shown to include dedicating the tax revenue for health-related programs, incorporating culturally sensitive messaging, and providing education on SSB consumption and poor health outcomes, while antitax messages primarily address government restriction of personal choice and negative economic effects on businesses.^17^ Like local SSB tax proposals in the cities of Richmond and El Monte, statewide SSB tax bills dedicated the tax revenue for health-related programs, clearly defined SSBs, and led with arguments linking SSB consumption to chronic health conditions. Common industry arguments focused on the disproportionate impact on low-income communities and job losses, which were also present in SSB tax debates in other countries.^56,57^

California’s experience with SSB taxation policies provides several lessons useful to policymakers and advocates in future efforts to promote a statewide SSB tax. Although a statewide SSB tax has failed in California to date, these instances of failure can pave the way for innovative policies to achieve passage. Future research should focus on gathering data on other important aspects of SSB tax policy debates, such as the appropriate use of revenues^58^ and collecting information on less publicly visible advocacy strategies like lobbying efforts. Recent evidence on local SSB taxes (Philadelphia, Seattle, and San Francisco) suggests the tax may have equitable impacts since they result in a sizeable net transfer of funds to lower-income populations.^14^ Moreover, this may be an effective evidence-based equity message to promote statewide legislation.

A key study strength is the study period since analyzing policy change should occur with data from at least a decade. Our findings provide a lens into the types of policy actors and advocacy activities that occurred in California over time. Inclusion of multiple data sources, including newspaper articles, is another strength, to triangulate data and as data repositories of policy activities.^59^ Limitations include use of data sources that might lack policy belief information. Additional data sources (i.e., alternative news sources like blogs or social media, stakeholder interviews) may have provided further information about policy beliefs or helped to identify any sub-coalitions or other coordinated advocacy activities that may not have been apparent in legislative bill or media documents. Second, it is possible that bills that were related to or impacted SSB taxes were missed like preemption law AB 1838 which focused on “grocery taxes.” Also, this study did not evaluate all components of the ACF, such as parameters and external subsystem events. The findings may not be generalizable to other states or policy debates since the coalition composition and actors might differ elsewhere. Finally, the study was limited to legislative bills and newspaper articles published between 1999- 2018, and, while this covers the period before preemption was in effect, other relevant bills may exist beyond this time period.

## Conclusion

This study uses document analysis to understand the composition and role of advocacy coalitions to support their policy positions at the state level. California’s failed attempts to enact statewide SSB tax legislation present an opportunity to identify actors involved in the policy debate and to examine their strategies and arguments to advance our knowledge of advocacy coalition behavior.

**So What?**

**What is Already Known About This Topic?**

Only local U.S. jurisdictions have successfully enacted sugar-sweetened beverage (SSB) taxes. Limited research exists on statewide SSB tax policy debates and the role of advocacy coalitions and their strategies in these debates.

**What Does This Article Add?**

Between 2001-2018, 11 SSB tax legislative bills were proposed in California. This study of legislative bills and media offers insight into the types of advocacy coalitions involved, their composition, and prevalent strategies and arguments used to advance policy positions.

**What are the Implications for Health Promotion Practice and Research?**

While an abundance of evidence exists on the effectiveness of SSB taxes, this public health coalition argument was not sufficient to advance an SSB tax in California. Preemption was a powerful tool leveraged by the industry coalition in opposition. We provide lessons learned from failed SSB tax legislation attempts which can inform future advocacy efforts.
